# Promoting Self-Regulatory Management of Chronic Pain Through Dohsa-hou: Single-Case Series of Low-Functioning Hemodialysis Patients

**DOI:** 10.3389/fpsyg.2019.01394

**Published:** 2019-06-20

**Authors:** Yutaka Haramaki, Russell Sarwar Kabir, Kazuaki Abe, Takashi Yoshitake

**Affiliations:** ^1^Department of Clinical Psychology, Hiroshima University, Higashihiroshima, Japan; ^2^Graduate School of Education, Hiroshima University, Hiroshima, Japan; ^3^Physiology and Pharmacology, Karolinska Institute, Solna, Sweden

**Keywords:** chronic conditions, chronic pain, self-regulation, Dohsa-hou, hemodialysis patients, pain management, quality of life, single-case design

## Abstract

Hemodialysis patients suffer from long-term pain that drains their energy and contributes to behavioral interference and other negative effects on their daily lives that result in or exacerbate functional limitations. In addition, they deal with dietary restrictions, symptoms such as itching, lack of energy, and psychological stressors like the loss of self-concept and self-esteem. Self-regulation involves the capacity to notice, inform, and modulate responses and behavior, and research indicates that it promotes rehabilitation in chronic pain patients. Research on the aspects of self-regulation afforded by the Japanese psychotherapy Dohsa-hou correspond to psychological processes tied to the sense of self-control that clients realize over their body movements. This study pilot tested a hospital-integrated implementation of Dohsa-hou relaxation tasks as a chronic pain management behavioral intervention for five female hemodialysis patients between the ages of 59–62 years. We conducted an ABABABA single-case design to compare baseline A-phases (treatment-as-usual: TAU) taken at recurring 1 week intervals (three sessions per week for a total of 4 weeks, 12 total recordings) with an intervention of Dohsa-hou B-phases every 4 weeks (three sessions per week for 12 weeks, 36 total recordings) over the span of 4 months to compare effectiveness. Visual Analogue Scale (VAS) pain scores between phases were taken and self-regulatory progress was tracked and summarized from a series of semi-structured interviews. Visual analysis of scores for each participant as single cases indicated decreases for the Dohsa-hou phase compared to baseline treatment-as-usual. As a result, participants reported using Dohsa-hou to reduce pain and experienced improvements in quality of life associated with greater self-regulatory capacity to attend to personal care and domestic activities. These preliminary findings suggest that Dohsa-hou body movement relaxation tasks were feasible as a coping skill in a hospital-integrated setting and at home and show promise for promoting quality of life vis-a-vis the management of severe and chronic bodily pain associated with end-stage renal disease and its treatment, particularly by improving aspects of pain-mediated self-regulatory fatigue.

## Introduction

Patients with chronic conditions are faced with the challenging prospect of comprehensively managing their disease and its symptoms. The discomfort of pain is a source of exacerbating demand under these circumstances. Pain becomes especially troublesome as patients are already making negotiations about the quality of their lives in the form of adjusting to the changes that are required to adhere to the treatment regimen of their disease. Psychologically, regaining control in the face of the adversity posed by the life event of chronic disease diagnosis, the onset of symptoms, and their treatment is a priority. While chronic conditions each have their own subset of challenges, the treatment of end-stage renal disease (ESRD) is characterized by not only the management of its symptoms, but also by the requisite difficulties imposed by its treatment, often in the form of chronic bodily pain. The primary aim of the current study was to develop a program to provide psychological support through relief from the bodily pain that patients experience under the duress of prolonged treatment for ESRD, and to pilot test and qualitatively evaluate its proof-of-concept for indications of changes in quality of life.

ESRD patients are treated with various modalities such as hemodialysis (HD), peritoneal dialysis (PD), and kidney transplantation. In an overview of the status of treatment for ESRD globally, Grassmann et al. (2005) reported comprehensive accounts of patient numbers, treatment modalities, and associated trends. Global ESRD patient numbers were estimated as encompassing 1,783,000 individuals, and among these 76.89% (1,371,000) received dialysis treatment (HD and PD) and 23.10% (412,000) received transplants. In the case of national statistics for Japan whose population was around 127 million at the time of the study, an estimated 261,000 individuals were ESRD patients, 95.02% (248,000) of which were dialysis patients and 4.98% (13,000) received transplants (Grassmann et al., 2005). In more recent accounts, ESRD patient numbers were estimated at 334,505 individuals for Japan in a survey by the Japanese Society for Dialysis Therapy at the end of 2017 (Nitta et al., 2018). Showing a similar pattern, 97.3% (325,415) of these individuals were HD patients and 2.7% (9,090) were continuous ambulatory peritoneal dialysis patients. Efforts to document the cumulative evidence of kidney transplant recipients by the Japan Academic Consortium of Kidney Transplantation cohort study estimated that 1,614 individuals received kidney transplants from 1990 to 2014 (Okumi et al., 2018). The Japan Organ Transplant Network (JOTNW) had registered 12,449 prospective recipients of kidney transplants in 2017 (Japan Organ Transplant Network (JOTNW)., 2019). Patients diagnosed with end stage renal failure are registered for kidneys, and JOTNW reported 182 kidney transplants had been conducted (Japan Organ Transplant Network (JOTNW)., 2019). In Japan, a major contingent of ESRD patients have received HD therapy for a prolonged period, and a majority of them have tended to be elderly. In fact, there were patients who have undergone HD for over 50 years in some cases. Due to clinical features from the relatively limited treatment options for ESRD, it is clear that long-term hemodialysis patients should be a focus for managed care in the context of Japan.

Psychosocial elements play a role in the adaptation to ESRD because undergoing dialysis treatment requires being physically connected to the HD machine three times a week over the course of a year, incurring a host of major stressors (Kaptein et al., 2010). Notably, patients are faced with lifestyle changes in the form of limitations to their food and fluid intake, symptoms such as itchiness and a lack of energy, and psychological components such as the loss of self-concept and self-esteem, feelings of uncertainty about the future, feelings of guilt toward family members, and other social consequences (Kaptein et al., 2010). Seen under the lens of chronic pain management and the integrative model of the biopsychosocial approach (Engel, 1977), HD patients often suffer from long-term pain that leaves them with little energy and negatively affects their quality of life. ESRD patients have poor quality of life (Feng et al., 2013) and the prevalence of acute and chronic pain in HD patients has been systematically reported to be up to 82 and 92%, respectively (Brkovic et al., 2016). In a previous study from Japan, hemodialysis patients suffering from chronic pain were shown to have pain-related constraints to the activities of daily living as a matter of disability, particularly in the realm of personal care and domestic activities (Haramaki and Nishi, 2012). Brkovic et al. (2016) concluded that improving quality of life and pain-related disability should represent a clinical and research priority and encouraged the nephrology community to promote pain management for HD patients.

Sauer et al. (2010) suggested that self-regulation theory (Carver and Sheier, 1982, 1998) is a useful integrative framework for understanding and treating chronic pain disorders. Behavioral and physiological measures indicate that individuals with chronic pain have lower self-regulatory capacity than those without it (Solberg Nes et al., 2009, 2010). Synthesizing clinical observations and experimental advances, Solberg Nes et al. (2009) proposed relationships between self-regulatory demands, executive functions, and self-regulatory fatigue. In their model, self-regulation demands require control to be exerted over emotions, thoughts, social relations, and behaviors. These relationships indicate that pain increases self-regulatory demands which in turn reduce executive functions. Case studies from Japan utilizing the body movement technique, *Dohsa-hou*, during HD therapy showed that HD patients with chronic pain reported reductions in psychological distress amenable to the experience of physical self-regulation (e.g., relaxation) gained from executing body movements with a sense of agency (Haramaki, 2011; Haramaki and Nishi, 2012). In the follow-up period, all patients reported maintaining their self-management by performing the body movements as an adaptive skill and experiencing improvements in daily life activities, such as housekeeping (Haramaki and Nishi, 2012). Together, observations gleaned from clinical settings indicated that Dohsa-hou might activate or otherwise influence an inherent motivation for patients to self-regulate, monitor, and maintain their health or activities tied to well-being.

In Japan, Dohsa-hou has been used for psychotherapy in various developmental and psychological conditions, including schizophrenia, depression, anxiety, and others (Naruse, 1997; Imura et al., 2016; Chervenkova, 2017; Fujino, 2017). Dohsa-hou facilitates behavioral change through body movements, feeling states of the body, and the experience of relaxation and embodied change as a means of therapeutic intervention and communication (Naruse, 1995; Fujino, 2012, 2017). Dohsa-hou was originally developed in Japan in 1966 by Gosaku Naruse from studies on the use of hypnosis to improve motor difficulties in children with cerebral palsy (Naruse, 1973). Naruse formulated the clinical observations of change into an underlying theory for Dohsa-hou, in which a coherence between the psychological and physiological process of movement is achieved when the client intends to move a body part, strives toward that goal, and realizes the movement they intended (Naruse, 1988, 1997; Kabir et al., 2018). Similar to other contemplative and bodymind approaches that target improvements in body movements, self-consciousness, mood, and decentering, Dohsa-hou increases awareness about the body through attention to bodily sensations and processes thought to confer motor resonance (Konno and Ohno, 1987; Dadkhah, 1998; Shirouzu and Koshikawa, 2011; Chervenkova, 2017; Kabir et al., 2018). The “Dohsa process” is designed to enhance a sense of agency over the body and is stipulated as a psychological activity. Comparably, self-regulation involves the capacity to exercise control and guide reactions, which are abilities essential to managing health outcomes and facilitating adaptive behavior. In this manner, the theory and practice of Dohsa-hou body movements and self-regulation overlap in the domain of motor control over body movements and health-related constructs for managing bodily signals and internal states (Chervenkova, 2017; Kabir et al., 2018).

A particularly salient subset of patients with chronic pain from a chronic condition are those who have undergone HD treatment for an extensive period and experience difficulties in their everyday life because of their pain. Such patients can become isolated in their social and familial roles as a result of the adaptive necessity to undergo HD therapy and deal with their chronic condition. Notably, patients have been learning adaptation skills for a long time. They often identify or make sense of their circumstances by finding roles and activities that they are able to do by themselves (e.g., housekeeping, do-it-yourself projects, volunteer activities, etc.) (Haramaki and Nishi, 2012). Patients who experience severe pain, long periods of inactivity, and more severe disabilities and symptoms in general are defined as lower-functioning patients (Friedberg et al., 2012), and might not be able to participate in group interventions. The variable nature of pain occurrence, intensity, and relief across time settings and limitations in their measurement precision makes the nomothetic approach to understanding clinical significance and determining therapeutic gain challenging (Morley and Williams, 2015). In turn, Morley and Williams (2015) proposed that greater attention should be paid to understanding *processes* within individuals in addition to the presence or absence of symptoms aggregated into descriptive categories designed for diagnosis. This approach prioritizes the individual and allows the therapist to recognize that the anxiety and distress associated with pain are not merely accounts of comorbidity but common outcomes of worry, frustration, losses of roles and pleasures, and fears about the future. Morley and Williams (2015) stipulated that, to improve outcomes for patients, distress and psychological disturbance should be interpreted under the context and the meaning of pain for the individual. Williams and Morley (2018) also put forth the notion that treatment for chronic pain may be gainfully tailored to fit patients toward increases in treatment efficiency, cost savings, expected improvement in treatment outcome, and patient satisfaction. Toward this end, effectiveness research is required to determine whether treatments are both feasible (e.g., exhibit procedural fidelity) and have clinical and social benefits that extend to other environments, especially in clinical practice (Williams and Morley, 2018). Therefore, leveraging the idiographic strengths of single-case designs would be especially suitable for understanding the processes of physical and psychological change, if any, that could occur from a pilot implementation under such circumstances (Tate et al., 2016; Williams and Morley, 2018) to determine degrees of applicability.

Pain elicits a response of increased muscle tension, which itself produces more pain, and contributes directly to secondary problems such as sleep disturbance and immobilization (Williams and Morley, 2018). Dohsa-hou contains some elements similar to the highly specific form of graded exposure *in vivo* to pain-related movements in the work by Vlaeyen et al. (2012), while also incorporating some elements attributed to third wave treatments (Morley and Williams, 2015; Chervenkova, 2017; Kabir et al., 2018). Dohsa-hou might endow sufferers of chronic pain with the opportunity to experience physical self-regulation and break the pain-tension cycle to handle self-regulatory demands or improve self-regulatory deficits (Solberg Nes et al., 2009).

Brkovic et al. (2016) noted that one of the most important parameters for evaluating patients' quality of life is through their degree of bodily pain, and that improving quality of life means improving quality of care by understanding and relieving bodily pain in HD patients. Therefore, the aim of this study is to investigate the effectiveness of a program targeting quality of life and self-regulation experiences by providing bodily pain relief and improvement from pain-related life interference as a way to augment the treatment regimen. This study designed and implemented a hospital-integrated program of body movement relaxation tasks to a sample of HD patients and investigated questions of effectiveness with single-case design and reporting. The study proposes that hemodialysis patients learn to manage their chronic pain on their own through the deliberate practice of movement tasks aimed at changing feeling states that can be applied upon the onset of pain and at home on an as-needed basis.

## Methods

### Impetus for the Study

Over a period of 3 years (2009–2011), the clinical staff members at the hemodialysis unit of a regional hospital in southern Japan participated in a stress-management program using Dohsa-hou relaxation tasks given by a psychotherapist with a supervisory license from the Japanese Association of Rehabilitation Psychology. The clinical psychologist demonstrated Dohsa-hou techniques for 5–10 min during staff meetings twice a month. The chief of staff requested that the clinical psychologist teach the clinical staff how they could apply Dohsa-hou to hemodialysis patients with chronic pain. All the staff agreed with this procedure: 7–10 patients had severe chronic pain, and the staff members were encouraged to give the patients the best care they could. In Japan, a HD patient normally receives dialysis treatment three times a week, with each session lasting 4 h. The staff at HD units provided patients with assistance not only in health matters but also with regard to matters of their daily activities. This facilitated the establishment of a cooperative relationship. It was observed that when patients suffer severe pain during dialysis treatment, analgesics are unable to provide sufficient relief. Thus, as recognized by the staff, the need to provide full treatment by alleviating pain for such patients served as an impetus for this study.

### Procedure and Setting of Dohsa Tasks

**Figure 1:**
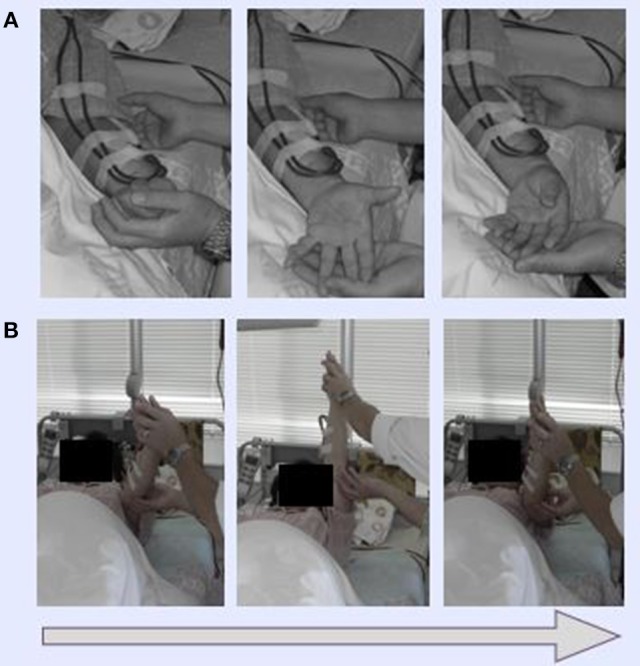
Demonstration of Dohsa movement tasks. **(A)** Dohsa tasks involved the clenching and opening of fists for the hands and bending back and forth for wrists. **(B)** Dohsa tasks also involved repeatedly raising and lowering the arms and shoulders along the body axis. All tasks were planned, and staff were given supervision for the psychological intervention by a licensed clinical psychologist certified by the Japanese Association of Rehabilitation Psychology.

**Figure 2:**
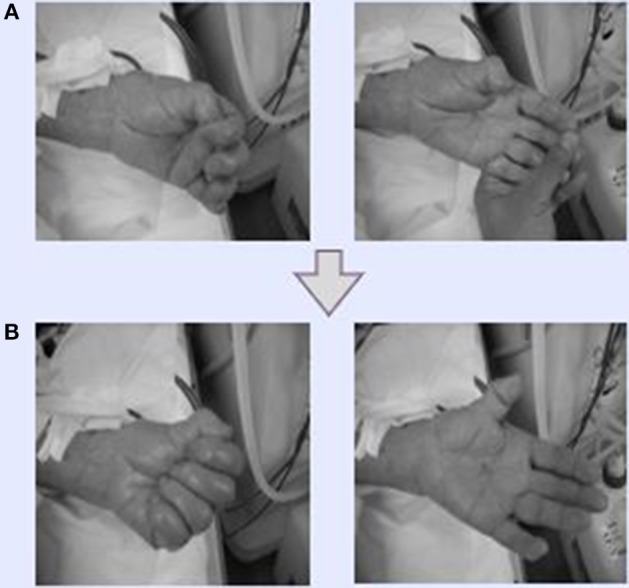
Effectiveness of Dohsa-hou hand movements. **(A)** Dohsa tasks conducted by a participant and supervised by medical staff during HD therapy. The staff clenched and opened their fists and bent their wrists back and forth repeatedly. **(B)** After the Dohsa-hou implementation, participants could move their hands and wrists by themselves. As a result, patients reported being able to bring and wash dishes in the kitchen and proactively perform housekeeping and daily activities at home.

HD patients typically suffer severe chronic pain at the shoulder, and in the neck and blood vessels, especially on the punctured side. In Dohsa-hou, there are two general types of tasks: relaxation and motor-action tasks. We selected relaxation tasks to be used on the patients in this study because they function to release excess muscle tension. The following Dohsa relaxation tasks were carried out by the patients in the supine position on a bed: (1) opening and closing the hand to achieve flexibility; (2) bending and stretching the wrist in a smooth movement; and (3) moving the arms and shoulders to relax stiffness in the shoulder and neck. The Dohsa-tasks performed and their effectiveness determined by phase are shown in Figures 1, 2.

The various steps in the Dohsa tasks were explained by means of diagrams for expanding participants' normal movement range. The Dohsa tasks were shown to be learned and replicable by the clinical staff (e.g., nurses). The staff was instructed to focus on the patient's body movement and the muscle tension they were undergoing; the staff members were also told to provide the patients with positive rather than negative feedback (e.g., “Very good, it's going smoothly. You're doing well, very well. OK. You can release your muscle tension”). Studies of Dohsa-hou in Japan have indicated that it is important to provide patients with positive feedback in regard to the awareness of their physical condition so as to improve their mental situation (Konno and Ohno, 1987; Dadkhah, 1998; Shirouzu and Koshikawa, 2011). When further questions were necessary, either the supervising clinical psychologist or the onsite clinical psychologist with a trainer license in Dohsa-hou who worked full time at the hospital visited the dialysis unit and supported or supervised the staff accordingly.

### Study Design

**Figure 3:**
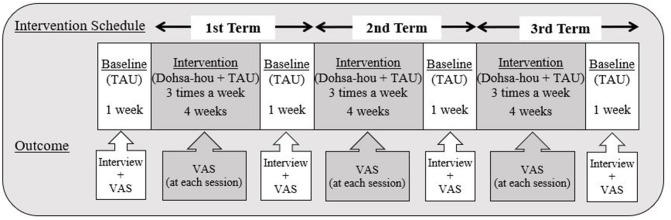
Implementation procedure and interview schedule. This figure displays the schedule of interviews and flow of data collection points for outcome measures. There were three total terms, and each term included 1 week of baseline TAU and 3 weeks of Dohsa-hou intervention, totaling 4 weeks per term. All participants received Dohsa-hou from medical staff when they complained about their pain at its peak. All participants reported their pain intensity along the Visual Analog Scale of pain (0–10) before dialysis treatment, pre-intervention, post-intervention (1 h after Dohsa-hou intervention) and at follow-up (1 month after research period). TAU, Treatment as usual; VAS, Visual Analogue Scale (for pain).

We employed an ABABABA single-case design. All baselines were 1 week treatment-as-usual (TAU) (12 recordings) A-phases and all intervention measurements were B-phases lasting for 4 weeks (36 recordings). The Dohsa-hou intervention was applied three times a week during HD therapy. To evaluate the effect of Dohsa-hou, VAS was used to measure pain intensity before and after the Dohsa-hou intervention. Drawing from time-based pacing used in cognitive behavioral therapy, a relatively brief restful pause is known to indicate that the patient is recovering to a predetermined safe amount of activity (Friedberg et al., 2012). This was the rationale for setting the TAU baseline A-phases as withdrawal periods. In addition, the general guideline for 4 weeks observations of treatment effects in studies of chronic pain was implemented (Gewandter et al., 2015). The total period of study was 16 weeks (16 weeks of study-sessions and 1 month for follow-up). All participants rested on their beds during HD therapy. The study schedule is depicted in Figure 3.

### Participants

#### Selection Criteria

The recruitment procedure was standardized along explicit inclusion criteria to target patients with chronic pain. We interviewed 69 patients (41 females) about their complaints of prolonged chronic pain. Nine patients (seven females) complained of chronic pain and seven patients (five females) gave their informed consent to participate in the study. Two male patients (71 and 69 years old) who underwent HD for a shorter period (0.3 and 0.8 years) and expressed apparent anxiety about HD from evaluation of the study, were excluded to maintain a focus on the effects of the intervention for long-term patients of severe pain and pain-related life interference associated with long-term hemodialysis treatment. This resulted in five patients who suffered from chronic bodily pain, all of whom had been recipients of hemodialysis treatment for over 10 years.

#### Participant Characteristics

Five women between the ages of 59 and 62 years (*M* age = 61 years old) who reported chronic pain that was particularly long-lasting and severe remained as the target participants for the present study. The patients underwent hemodialysis treatment over a long period (minimum = 16 years; maximum = 34 years; mean = 28 years). The patients were unable to do any work, having been inactive and housebound for approximately 5 years or more because of their condition. They complained of chronic pain (shoulder stiffness and pain in the vein that was punctured by the needle) during dialysis and often had to halt the treatment because of the severity (shoulder stiffness or vein pain), and this also caused difficulties in their daily lives. They took analgesics, but the effectiveness was variable (Table 1; treatment as usual). It should be noted that the ability of more severely ill patients (e.g., those with disabilities and largely homebound) to perform therapeutic tasks such as undergoing 30 min of additional exercise or attending counseling sessions once a week may be limited due to debilitating post-exertional pain (Nijs et al., 2008). Recent clinical studies into the condition of chronic pain patients have often used pain-related questionnaires (McCracken and Velleman, 2010; Solberg Nes et al., 2010; Wetherell et al., 2011; Wicksell et al., 2011; Hayes et al., 2013). In order to investigate an improvement in quality of life from changes in pain experiences, the patients were interviewed about their subjective experiences of pain and its interfering effects on their daily life. None of the patients had diagnoses of psychiatric illness (e.g., schizophrenia and depression).

**Table 1:** Baseline characteristics and treatment-as-usual (TAU) for the study participants.

**ID**	**Age (year)**	**Sex**	**Dialysis period (year)**	**Chronic pain**	**Analgesic(s)**	**Physical therapy**	**Complaint(s)**
A	60	Female	16.8	Shoulder, knee	Compresses	Xenon	Difficulty with housekeeping
B	62	Female	27.8	Shoulder, knee	Loxoprofen compresses	Xenon	Difficulty with housekeeping Difficulty and disability with home activity
C	59	Female	28	Shoulder, back, chest	Loxoprofen compresses	Xenon	Difficulty with housekeeping Difficulty and disability with home activity Disability of body movement
D	62	Female	34.3	Shoulder, knee, back, hip joint	Loxoprofen compresses	Xenon Hot pack Exercise therapy	Difficulty with housekeeping Disability of home activity
E	62	Female	34.8	Shoulder, knee, back	Loxoprofen compresses	Xenon Hot pack Exercise therapy	Difficulty with housekeeping Disability of home activity Difficulty with sleeping during dialysis

*All participants received their usual care for existing pain, although its effectiveness was uncertain. All staff agreed that patients needed pain care additional to their usual treatment*.

*Xenon light refers to permeable, near-infrared wavelengths used for light therapy for living organisms. The use of photodynamic therapy relieves pain by improving blood flow*.

### Ethical Approval

Hemodialysis nursing staff and clinical psychologists set the study protocol. The head of hospital, vice-head of hospital, and nursing supervisor of the hospital approved the study protocol instead of an institutional review board. All the participants gave their written informed consent and voluntarily took part in the study. The study was retrospectively reviewed for compliance and approved in line with protocols from the ethical research committee of the Graduate School of Education, Hiroshima University. This review was necessary for reasons of institutional and affiliation change on the part of the principal investigator since the time of the study, and not due to ethical issues in the protocol that occurred before, during, or after the study implementation.

### Outcome Measures

Avoiding additional sources of physical or psychological burden for the participants was a priority in the design of the implementation. Therefore, in lieu of other invasive evaluation techniques, this study focused on two outcome measures to prioritize idiographic assessment: VAS pain scores and reports from semi-structured interviews.

The VAS pain scores included pain intensity levels, physical activity, quality of life, and life activity (or life interference). The VAS used response scales for a single item with a horizontal form: a 100 mm line anchored upward by “pain as bad as it could be” (10) and anchored downward by “no pain” (0); the patient was instructed to mark any point on the VAS continuum corresponding to their present pain intensity (Jane et al., 2011; Morley, 2018). VAS pain scores were evaluated analogically by all the participants before dialysis treatment, at 1, 2, 3, and 4 h after starting HD therapy, and after treatment. When staff conducted the Dohsa-hou intervention, VAS pain scores were measured pre and post-intervention.

The interview schedule is depicted in Figure 3. All participants completed the reporting at each TAU A-phase (1–3, 16–18, 31–33, and 46–48 sessions) and at follow-up (1 month after the study). All of the participants were given a semi-structured interview. The interview consisted of three main questions about the persistence of pain, the effects of Dohsa-hou, and daily life activities (especially including using the relaxation tasks in their normal daily lives). In line with the priorities of idiographic assessment that frame patient reporting of problems as the target of therapeutic change (Morley, 2018), we decided to consider patient-generated complaints and issues for regulating personal care and domestic activities (housekeeping and daily activities) as outcomes for examination.

### Intervention

During the course of the study, all participants continued receiving their usual health care (TAU), including treatment for pain and other medical conditions, such as hypertension. The Dohsa-hou intervention was conducted on an individual basis and consisted of 36 sessions for each patient. The Dohsa-hou intervention schedule consisted of three terms. Complaints of pain from the five participants usually occurred around 2 h after starting HD. We conducted the Dohsa-hou tasks when participants complained of their pain. If participants did not complain of pain, we implemented Dohsa-hou at around the 2 h mark. Each intervention term lasted a total of 5 weeks, comprising 4 weeks of Dohsa-hou intervention and 1 week of TAU (see Figure 3).

## Data Preparation

### VAS Scores

**Figure 4:**
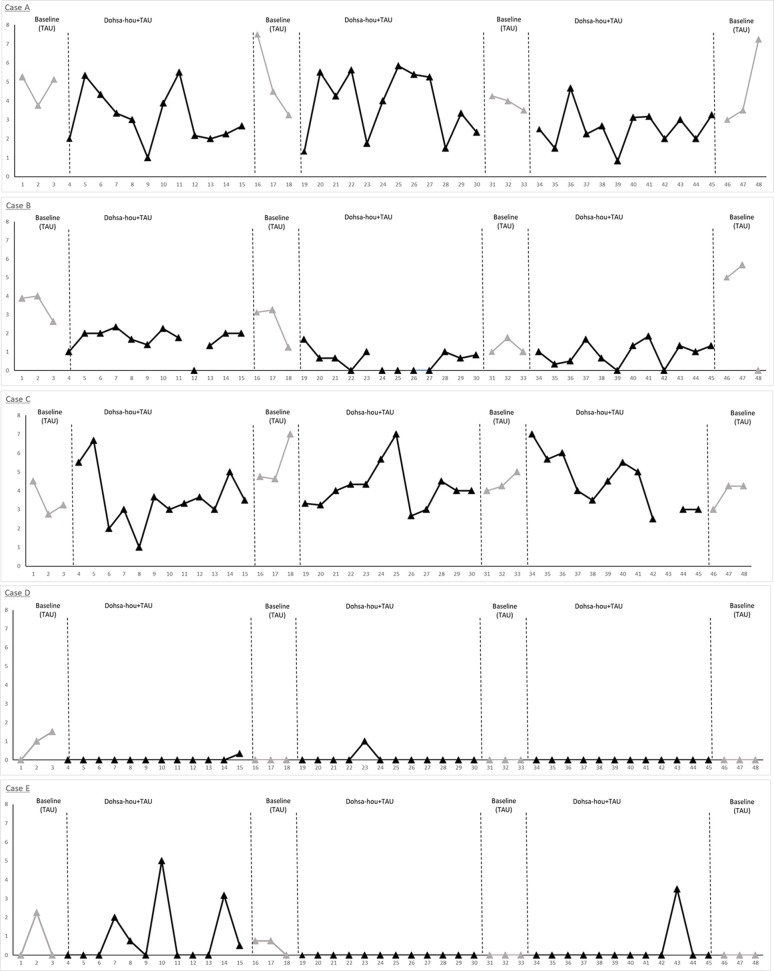
Single-case data plots of the ABABABA design for each participant. VAS pain scores, measured five times during HD therapy, were averaged from hourly scores obtained at TAU and intervention phases. The VAS pain scores ranged from 0 to 10. Case A and C reported higher scores indicating severe pain which remained throughout the study period. Pain intensity for Case B, D, and E gradually reduced after 19 sessions. Case A and B had shunt occlusion issues at session 46 that kept them from receiving sufficient HD therapy for the final two sessions.

The VAS pain scores were averaged at 2, 3, and 4 h, and compared between intervention and TAU. The VAS pain scores were divided into two groups: one group consisted of pre-intervention values; the other consisted of post-intervention values. Participants received intervention at peak times of pain. Pre-intervention VAS pain scores were averaged from pre-dialysis treatment to before intervention. Post-intervention VAS pain scores were averaged from after intervention to 4 h after dialysis treatment. The VAS pain scores were analyzed for differences between pre-intervention and post-intervention. Visual analysis of the scores was adopted to clarify the effectiveness of the Dohsa-hou B-phases compared to the TAU baseline A-phases within the single-case design. Data plots for the series of sessions of each case are depicted in Figure 4. In addition, percentage-below-the-median analysis was performed as single case estimates of effect size by comparing median VAS scores from baseline TAU and Dohsa intervention phases, as demonstrated in Table 3 (Bulté and Onghena, 2013; Morley, 2018).

### Interview Data

All participants were interviewed at the end of the first, second and third terms within the baseline TAU A-phases. The interview questions were structured to determine applied effectiveness of pain management (Haramaki and Nishi, 2012; Williams and Morley, 2018). Each case was qualitatively assessed for categories of pain management effectiveness (pain reduction or relief experiences) and indicators of behavioral regulation as theorized by Solberg Nes et al. (2009). Two clinical psychologists familiar with the cognitive, emotional and behavioral aspects of self-regulation in clinical and laboratory settings determined the fit of the interview content for each of the categories and subcategories by rating affinity and inclusion with a “Yes” or “No.” The fit of correspondence between the interview content to the domains of interest are depicted in Table 2.

**Table 2:** Ratings for categorical inclusion of interview data indicating qualitative effectiveness of Dohsa-hou for self-regulation under chronic pain from hemodialysis.

**Participants**	**A**					**B**					**C**					**D**					**E**				
	**BL**	**1st**	**2nd**	**3rd**	**FU**	**BL**	**1st**	**2nd**	**3rd**	**FU**	**BL**	**1st**	**2nd**	**3rd**	**FU**	**BL**	**1st**	**2nd**	**3rd**	**FU**	**BL**	**1st**	**2nd**	**3rd**	**FU**
**EFFECTIVENESS**																									
**Pain reduction or experiences of pain relief**																									
Pain reduction	No	Yes	Yes	Yes	Yes	No	Yes	Yes	Yes	Yes	No	Yes	Yes	Yes	Yes	No	Yes	Yes	Yes	Yes	No	Yes	Yes	Yes	Yes
Reducing analgesics	No	No	No	Yes	Yes	No	Yes	Yes	Yes	Yes	No	No	Yes	Yes	Yes	No	Yes	Yes	No	No	No	–	–	–	–
Relaxation experience	No	No	Yes	Yes	Yes	No	No	Yes	Yes	Yes	No	Yes	Yes	Yes	Yes	No	Yes	Yes	Yes	Yes	No	Yes	Yes	Yes	Yes
Positive feelings toward or within the body	No	Yes	Yes	Yes	Yes	No	No	Yes	Yes	Yes	No	Yes	Yes	Yes	Yes	No	Yes	Yes	Yes	Yes	No	Yes	Yes	Yes	Yes
Bringing a sense of security	No	No	No	No	Yes	No	No	No	No	No	No	No	No	No	No	No	No	No	No	No	No	No	No	Yes	Yes
Total present	0	2	3	4	5	0	2	4	4	4	0	3	4	4	4	0	4	4	3	3	0	3	3	4	4
**Pain management**																									
Analgesics	Yes	Yes	Yes	Yes	Yes	Yes	Yes	Yes	Yes	Yes	Yes	Yes	Yes	Yes	Yes	Yes	Yes	Yes	Yes	Yes	Yes	Yes	Yes	Yes	Yes
Physical therapy	Yes	Yes	Yes	Yes	Yes	Yes	Yes	Yes	Yes	Yes	Yes	Yes	Yes	Yes	Yes	Yes	Yes	Yes	Yes	Yes	Yes	Yes	Yes	Yes	Yes
Engaging with Dohsa-hou by myself	No	Yes	Yes	Yes	Yes	No	No	Yes	Yes	Yes	No	Yes	Yes	Yes	Yes	No	Yes	Yes	Yes	Yes	No	Yes	Yes	Yes	Yes
Self-management for pain through Dohsa-hou	No	Yes	Yes	Yes	Yes	No	No	Yes	Yes	Yes	No	Yes	Yes	Yes	Yes	No	Yes	Yes	Yes	Yes	No	No	Yes	Yes	Yes
Using both analgesics and Dohsa-hou	No	Yes	Yes	Yes	Yes	No	No	Yes	Yes	Yes	No	Yes	Yes	Yes	Yes	No	Yes	Yes	Yes	Yes	No	No	Yes	Yes	Yes
Total present	2	5	5	5	5	2	2	5	5	5	2	5	5	5	5	2	5	5	5	5	2	3	5	5	5
**Behavioral regulation (deficits)**																									
Regulating and controlling daily activities and behavior	Yes	No	No	No	No	Yes	–	No	No	No	Yes	No	No	No	No	Yes	Yes	No	No	No	Yes	Yes	Yes	No	No
Staying involved in treatment and appointments	–	–	–	–	–	–	–	–	–	–	–	–	–	–	–	–	–	–	–	–	–	–	–	–	–
Act in socially accepted and appreciated manners	–	–	–	–	–	–	–	–	–	–	–	–	–	–	–	–	–	–	–	–	–	–	–	–	–
Deficits in planning and decision making	–	–	–	–	–	–	–	–	–	–	–	–	–	–	–	Yes	No	No	No	No	Yes	Yes	Yes	No	No
Difficulties overriding automatic processes	Yes	No	No	No	No	Yes	No	No	No	No	Yes	No	No	No	No	Yes	Yes	No	No	No	Yes	Yes	Yes	No	No
Passive coping, avoidance, disengagement	Yes	–	–	–	–	Yes	–	–	–	–	–	–	–	–	–	–	–	–	–	–	Yes	Yes	Yes	No	No
Avoidance of physical exercise	Yes	–	–	–	–	Yes	–	No	–	–	–	–	–	–	–	Yes	–	–	–	–	Yes	Yes	Yes	–	–
Difficulties overriding urges, inactivity, fatigue	Yes	No	No	No	No	Yes	No	No	No	No	Yes	No	No	No	No	Yes	Yes	No	No	No	Yes	Yes	Yes	No	No
Total present	5	0	0	0	0	5	0	0	0	0	3	0	0	0	0	5	3	0	0	0	6	6	6	0	0

*Effectiveness is conceptualized in terms of pain relief or reduction and pain management. Criteria for behavioral regulation as a self-regulatory domain was interpreted in accordance with the framework of chronic pain from self-regulatory demands and deficits conceptualized by Solberg Nes et al. (2009). Semi-structured interview data were evaluated under these categories*.

## Results

### Background Case Characteristics

The patients had been making an effort to adapt to their severe illness (end-stage renal failure) over a long period. The participants reported having experienced severe pain during dialysis and at home for the previous 1–3 years. All the participants complained of difficulty performing everyday activities (e.g., housekeeping and cooking at home) because of their pain. Previous studies found that HD patients with chronic pain held their role as a member of their family with meaningful contributions to domestic activities as an important aspect of their self-concept under the circumstances of dealing with their chronic condition and its treatment (Haramaki, 2011; Haramaki and Nishi, 2012). All the participants received pain medicine and physical therapy: five participants used compresses that included analgesics; four were treated with loxoprofen; five were treated with xenon (physical therapy given by orthopedics) and hot-pack treatments; and two received exercise therapy (Table 1). It was determined that the patients were already experiencing high levels of self-regulatory fatigue, and hence it was expected that they would soon have even less capacity to do housekeeping.

### Indicators of Effectiveness From Integration With the Hospital Setting

Dohsa-hou was applied by the staff to the patients on their beds when they complained of severe pain during dialysis treatment. It was observed that when the patients suffered intense pain, they had difficulty expressing themselves and were unable to describe their condition. However, if the staff gave the patients their full attention, the patients became better able to compose themselves and explain how they felt. However, because of the great demands on their time, staff members were formerly unable to devote too much time to each individual patient. In addition, many of the staff had no experience developing pain-related communication skills. In this study, both the staff and patients had the opportunity to acquire such skills through the process of giving and obtaining VAS pain scores, the application of Dohsa-hou tasks, and participating in the associated interviews. As a result of carrying out the Dohsa tasks with the help of clinical staff, the patients were able to release excessive muscle tension in the wrist, upper arm, shoulder, and neck by themselves.

### Pain Reduction Effectiveness of Dohsa-hou Across Chronic Pain Patients

Pain management effectiveness was demonstrated from visual analysis inspection (Figure 4). Single-case data plots of VAS pain scores are provided in Figure 4, and percentage-below-the-median analysis was performed for single case estimates of effect size by comparing baseline TAU and Dohsa intervention phases (Table 3). These results suggest that the Dohsa-hou relaxation task intervention phase contributed to pain alleviation among the participants. These effects of pain reduction continued for 16 weeks (~4 months) and were thus not temporary. VAS pain scores for the third pre-intervention were higher than for the other pre-intervention scores, but not considerably so.

**Table 3:** Single-case percent below the median analysis for VAS scores between treatment-as-usual baseline A-phases and Dohsa-hou intervention B-phases.

**ID**	**A-1**	**B-1**	**No. sessions below median**	**Percent below median (%)**	**A-2**	**B-2**	**No. sessions below median**	**Percent below median (%)**	**A-3**	**B-3**	**No. sessions below median**	**Percent below median (%)**	**A-4**
A	5.13	2.835	10/12	83.3	4.5	4.125	7/12	58.3	4	2.585	11/12	91.6	3.15
B	3.875	2	11/11	100	3.125	0.667	10/10	100	1	1	5/12	41.6	5.33
C	3.25	3.416	6/12	50	4.75	4	10/12	83.3	4.25	4.5	5/11	45.5	4.25
D	1	0	12/12	100	0	0	11/12	91.6	0	0	12/12	N/A	0
E	1.125	0	8/11	72.7	0.75	0	12/12	100	0	0	11/12	91.6	0

*The median refers to the median of averaged VAS scores from each session in all phases. The scores can be cross-referenced with the VAS changes depicted in Figure 4*.

### Case-By-Case Effectiveness of Dohsa-hou From VAS Scores and Interview Data Including Follow-Up

After each Dohsa-hou session in the intervention periods, all participants reported freely about their pain and Dohsa-hou, as well as in the semi-structured interviews taken during TAU periods (TAU: analgesics and physical therapy). In terms of sequences completed, all five participants were able to complete almost all of the sessions to undergo the Dohsa tasks except at four data collection points (Case B, one missing session in the first term; Case C, one missing session in the first and third term; Case E, one missing session in the first term; for details, see Figure 4 and Table 3), indicating low attrition of task implementation and a degree of procedural fidelity throughout the period of study.

Ratings for categorical inclusion of interview data indicating qualitative effectiveness of Dohsa-hou for self-regulation under chronic pain from hemodialysis are depicted in Table 2. The first two sections of Table 2 show the effectiveness of each participant's pain reduction and relief experiences and pain management behaviors. Across participants, qualitative change indicating pain reduction or relief change occurred after first term of Dohsa-hou session and that criterion of effectiveness was maintained until follow-up. In terms of pain reduction and relief experiences, all participants reported experiencing pain reduction from the first term to follow-up, and points of change for experiencing relaxation and positive feelings toward the body occurred from the first term for three participants and the second term for two participants.

The bottom section of Table 2 depicts the effectiveness of promoting self-management according to itemized aspects of behavioral regulation from the interview content. The onset of effectiveness of the intervention in this domain was more variable and required more detailed case-by-case considerations which are provided in the following section.

#### Case A

As can be seen in Figure 4, Case A indicated elevated pain scores and unstable pain conditions during the study period, but also demonstrated incremental reporting of pain relief and pain management experiences. Along the column of Table 2, Case A mentioned experiencing pain reduction and positive feelings toward their own body beginning in the first term and lasting throughout the study. Also, Case A reported experiencing relaxation and reducing analgesics at the second and third term. At follow-up, Case A reported a sense of security. For pain management, Case A mentioned engaging in not only the use of analgesics and physical therapy but conducting Dohsa-hou by themselves as a means of self-management for pain and using both analgesics and Dohsa-hou from the first term until the follow-up term. In terms of indicators for behavioral regulation, all of the self-regulatory deficits Case A reported at baseline session (e.g., deficits in regulating and controlling daily activities and behavior, inactivity, and fatigue) were not mentioned from the first term until the follow-up term. In summary of the results of visual analysis depicted in Table 3, 83.3% of the VAS scores from Case A for the Dohsa-hou sessions were below the median baseline for the first term, 58.3% under the baseline median for the second term, and 91.6% for the third term. From inspection of the VAS score medians for the baseline to follow-up A-phases, Case A exhibited a general trend of decreasing reports of pain intensity over the period of study, with a pattern of smaller median VAS pain scores in the B-phases than A-phases.

#### Case B

For VAS pain scores, Case B showed trends toward alleviating their pain from the first term (Table 3). However, at the follow-up term, the pain score increased. As can be seen in Table 2, Case B reported pain reduction, reducing analgesics from first term, and the experience of relaxation and positive feelings toward their own body from second term. However, a sense of security was not reported during intervention. For pain management, Case B mentioned engaging in not only analgesics and physical therapy, but also that they conducted Dohsa-hou by themselves as self-management for pain and used both analgesics and Dohsa-hou from second term until the follow-up term. For behavioral regulation, most of the deficits Case B reported at the baseline session were not mentioned from the second term until the follow-up term. In summary of the results of visual analysis depicted in Table 3, 100% of the VAS scores from Case B for the Dohsa-hou sessions were below the median baseline for the first term, 100% for the second term, and 41.6% for the third term. From inspection of the VAS score medians across phases, Case B exhibited longitudinally variable differences in reports of pain intensity.

#### Case C

Referring to Figure 4, Case C indicated elevated pain scores and unstable pain conditions during the research period. Case C reported pain reduction, relaxation experience, positive feelings on own body from first term, and reported reducing analgesics from second term. However, a sense of security was not reported during intervention (Table 2). For pain management, Case C also mentioned engaging in not only analgesics and physical therapy but using Dohsa-hou by themselves for self-management of pain and using both analgesics and Dohsa-hou from the first term until the follow-up term. Also, for behavioral regulation, all of the deficits Case C reported at the baseline session were not mentioned from the first term until the follow-up term. In summary of the results of visual analysis depicted in Table 3, 50% of the VAS scores from Case C for the Dohsa-hou sessions were below the median baseline for the first term, 83.3% under the baseline median for the second term, and 45.5% for the third term. From inspection of the VAS score medians for the baseline to follow-up A-phases, Case C exhibited variable reports in pain intensity over the period of study.

#### Case D

Case D showed trends toward alleviating their pain from the first term (Figure 4). Shown in Table 2, Case D reported pain reduction, reducing analgesics, experiencing relaxation, and having positive feelings toward their body from the first term. However, reducing analgesics was not reported from third term and a sense of security was not reported during the intervention. For pain management, Case D engaged in not only analgesics and physical therapy, but self-management for pain through Dohsa-hou, as well as using both analgesics and Dohsa-hou from the first term until the follow-up term. In terms of behavioral regulation, most of the deficits Case D reported at the baseline session were not mentioned from the second term until the follow-up term. In summary of the results of visual analysis depicted in Table 3, 100% of the VAS scores from Case D for the Dohsa-hou sessions were below the median baseline for the first term, 100% under the baseline median for the second term, and no reports of pain intensity at all for the sessions of the third term in either of the phases. From inspection of the VAS score medians for the baseline to follow-up A-phases, Case D exhibited a general trend of decreasing reports in pain intensity over the period of study.

#### Case E

Case E showed trends toward alleviating their pain from the first term onward (Figure 4). Depicted in Table 2, Case E mentioned experiencing pain reduction, relaxation, and positive feelings toward their body from the first term. Also, Case E reported a sense of security from third term. For pain management, Case E mentioned engaging in not only analgesics and physical therapy, but also Dohsa-hou as self-management for pain conducted by themselves and using both analgesics and Dohsa-hou from the second term until the follow-up term. For changes in behavioral regulation, most of deficits Case E reported at baseline session were not mentioned from the third term until the follow-up term. In summary of the results of visual analysis depicted in Table 3, 72.7% of the VAS scores from Case E for the Dohsa-hou sessions were below the median baseline for the first term, 100% under the baseline median for the second term, and 91.6% for the third term. From inspection of the VAS score medians for the baseline to follow-up A-phases, Case E exhibited a general trend of decreasing reports pain in intensity over the period of study.

## Discussion

The aim of this study was to investigate the proof-of-concept and effectiveness of a hospital-integrated program of Dohsa-hou movements for indicators of bodily pain relief, improvement of pain-related life interference as indicators of quality of life and self-regulatory change among low-functioning patients undergoing long-term hemodialysis treatment.

### Reduction in Chronic Pain During Dialysis Treatment

With regard to our first research question that Dohsa-hou will contribute to a reduction in persistent chronic pain during dialysis treatment, the patients reported decreased pain intensity levels in terms of VAS scores and qualitative changes in behavioral regulation, although with varied effects by term. The results of visual analysis depicted in Figure 4 tentatively suggest that the Dohsa-hou phase was effective in promoting pain reduction, as Case B, D, and E exhibited decreases in their pain levels during the course of the study. Case A and C demonstrated more unstable VAS scores in keeping with the nature of their pain conditions; notably, these cases had severe pain associated with multiorgan failure. After the 46th session, Case A and B were diagnosed with shunt vessel occlusion (Figure 4, see Case A and B). Owing to the precariousness of the symptom, they could not receive HD therapy sufficiently at that time. After diagnosis, the two participants were able to receive adequate treatment for the shunt trouble. In spite of this, comparing the intervention and TAU phases, the results of single-case series reports of qualitative interviews taken during the intervention and at follow-up (Tables 2, 3) together with VAS score change comparisons suggest that the Dohsa-hou intervention phase appeared to contribute to chronic pain reduction and self-management behavior for particularly three out of the five cases.

### Improvements in Pain-Related Interference

With respect to our second research question, Dohsa-hou was associated with improvements from pain-related life interference via reports of decreased emotional distress and improved activity levels, self-management ability, and quality of life for the low-functioning HD patients with chronic pain conditions. Table 2 shows qualitative points of change as improvements in pain experience, pain-related activities, regulatory behavior, and lessened interference with personal care and domestic activities. Notably, many of these indicators of effectiveness often had an early onset across cases and were maintained from the first term to follow-up. These results indicate that Dohsa-hou was associated with improvements in pain-related life interference in terms of increases in activity levels, self-management ability (coping with Dohsa-hou), and daily life improvements. In this manner, Dohsa-hou established a proof-of-concept for application and promoted aspects of self-regulation, mainly in the realm of behavioral regulation, for improving pain-related life interference.

### Promoting Quality of Life Through Self-Regulation via Dohsa-hou

In addition to lessening pain-related interference, it is important to offer coping strategies that increase self-regulatory capacity as psychological flexibility (Davey, 2016). Self-regulation theory (Carver and Sheier, 1982, 1998) underlies these self-care and coping skills as a framework for understanding and treating chronic pain conditions (Sauer et al., 2010). Self-regulation theory is based on behavioral theory where the focus is on the self and self-regulatory resources. Solberg Nes et al. (2009, 2010) found that chronic pain patients were more vulnerable to self-regulatory fatigue than matched controls. In other studies, the impact of chronic pain conditions on self-regulatory efforts was found to be mediated by pain, not by any other factors (Solberg Nes et al., 2010). Since physical self-regulation aims to redirect self-regulatory efforts toward regulation of the autonomic nervous system and emotional responses, it is useful in managing chronic disorders, and observed benefits may be easily extended to other chronic pain conditions (Carlson et al., 2001).

Dohsa-hou is a method that leverages the physiological functions of body movement to exert a direct effect on a person's psychological activity. The sense of body control in this paradigm arises from finding, feeling, and witnessing a coherence between an intended movement and a synchronous result (Chervenkova, 2017; Kabir et al., 2018). When a client acquires new body movement skills, the therapist can reasonably infer that efforts are being volitionally applied toward facing real-life psychological problems (Naruse, 1988; Haramaki, 2011; Haramaki and Nishi, 2012). Many aspects of the mechanism responsible for the clinical effects are still unclear, but some studies have indicated that the act of accomplishing Dohsa tasks contributes to body awareness that facilitates an enhanced sense of self-control (Konno and Ohno, 1987; Dadkhah, 1998; Hoshikawa, 1998; Shirouzu and Koshikawa, 2011; Fujino, 2017; Kabir et al., 2018). All participants were receiving intensive HD treatment and experienced limitations in their daily lives due to chronic pain for long periods. They were adapting to these chronic conditions characterized by low-functional status, and restricting their activity in accordance with their symptoms, pain, and other factors. Dohsa-hou encouraged all participants to engage or re-engage with personal care and domestic activities (Tables 2, 3) while staying at home in the form of washing dishes, hanging up washed-clothes to dry, and decreasing the use of analgesics. While two of the cases exhibited instances of unstable VAS reporting, turning points in the analysis of the data from semi-structured interviews especially suggested that rehabilitated ability to commit to desired activities were reflected in the time course of the intervention. In this way, the results suggest that the Dohsa-hou phases conferred some effectiveness toward pain reduction (Williams and Morley, 2018) by incrementally and continuously promoting behavioral aspects of self-regulation (Tables 2, 3).

In terms of the significance of these findings to research on Dohsa-hou, they add to the evidence base that suggests the psychotherapy overlaps or interfaces with mechanisms of physical self-regulation (Sauer et al., 2010), especially in the narrative sense. Descriptive comparison between clinical Dohsa-hou and the narrowed repertory of movements explicitly targeting and augmenting “self-activeness” known as Self-Active Relaxation Therapy supports this notion (Kabir et al., 2018). The focus on activity management, relaxation, and behavioral adjustment (Davey, 2016) in terms of personal care and domestic activities framed the goals of therapy under their terms and allowed for a more fine-grained evaluation of expected benefit or change. Similar to its roots in developmental conditions and movement disorders, physical regulation via Dohsa-hou might be especially equipped to target issues in functional status because the frame for goals is anchored in the simple performance of movements, in witnessing the self as the agent of change, in redirecting attention to the body, and in viscerally perceiving the accompanied experience of relaxation. In this manner, it might also be reasonable to assume that patients undergoing dialysis exhibit distress or fear of movement associated with their treatment, as body tension and movement restriction might be expected while being attached to the HD machine.

The present study did show some inconsistencies in the points of change between terms for two of the cases. Cases A and B seemed to acquire self-regulation abilities during the Dohsa-hou session compared with TAU (Table 2 and Figure 4), but Cases A and B were less conclusive about the treatment effect due to fluctuations in VAS pain scores. This indicates a discrepancy between the comments from interviews indicating positive psychological change from self-regulation and VAS score results. One reason might be traced to the aforementioned experiences of severe pain between the weeks of 46 and 48 weeks that were documented to be due to issues with shunt occlusion (Figure 4). However, this could also be due to limitations in the study design or interview methodology to pinpoint points of change or account for treatment expectancy (see Limitations section). While this study employed these methods to keep the study burden as low as possible in an effort to facilitate high-fidelity implementation, this limitation underscores the need for future studies to extend beyond a proof-of-concept and address the treatment effect against a randomized control group. These points notwithstanding, it can reasonably be inferred as a preliminary finding that the Dohsa-hou phases were attributed with the promotion of self-regulation and a degree of quality of life improvement in the place of severe pain and fatigue, otherwise the results from the interview questions would have been chiefly comprised of continuous reports of severe pain and patterned idiographic indications of incremental VAS pain score reduction over the course of the study would not have been observed. While more clearly evident in the other three cases, these findings remain tentative as despite the use of longitudinal clinical observation, our results chiefly represent a proof-of-concept that should be carefully interpreted until the implementation is compared with stronger designs that can address possible risks of bias.

While further examination is necessary to parse the treatment effect more precisely, we surmise that one element in the mechanism of psychological change or direct contribution from physical movements in the Dohsa-hou framework to pain reduction might be similar to aspects of graded exposure *in vivo* for movements that patients might find threatening due to being recipients of intensive treatment (de Jong et al., 2005; Morley and Williams, 2015). In contrast, or in conjunction with this theoretical assertion, the relaxation element of Dohsa-hou might function as a response modulator under the umbrella of stress coping or emotion regulation (Gross and Thompson, 2007), or recruit awareness or changes in interoceptive attention tendencies (Kabir et al., 2018) that extend to pain regulation, but further research is necessary. The constructs of pain vulnerability antecedents and their consequences might provide the necessary theoretical framework or otherwise usefully clarify the contribution of Dohsa-hou to pain-related emotion regulation (Koechlin et al., 2018). Nevertheless, the approach of Dohsa-hou, like many others, appears to raise awareness about the body, and in this study, patients were able to overcome elements of their pain-related interference and functional limitations by recognizing and demonstrating some especially behaviorally-oriented regulatory capacity changes on the spectrum of adaptive behavior.

### Feasibility of Dohsa-hou Implementation for Pain Management

The strength of this study lies in its ability able to provide a new and accessible pain management skill for HD patients with chronic pain. To the best of our knowledge, this is the first program of its kind for hemodialysis patients in Japan. Participants demonstrated indications that they were able to acquire self-regulation skills over the period of 16 weeks until follow-up (1 month after third term: Tables 2, 3). All participants could notice a reduction in pain (changes in VAS pain score; Figure 4) and improvements in daily-life interferences as indicators of quality of life change through self-regulation activities that were associated with the Dohsa-hou phases (Tables 2, 3). This seems to indicate that as the patients underwent Dohsa-hou, they adopted the relaxation process as a new way of coping with their chronic pain. In this way, efforts to achieve relaxation allowed the participants to break the pain-tension cycle (Williams and Morley, 2018) and vicious cycle of self-regulatory fatigue, specifically by acquiring the concrete relaxation tasks as new pain management skills to supplement their day-to-day lives.

In the present study, all of the participants demonstrated an ability to monitor and attend to their body more effectively by performing the Dohsa tasks and taking part in the semi-structured interviews (Table 3). Training in communication skills can be conducted with patients and staff in a general setting in terms of bedside psychotherapy (Griffith and Gaby, 2005) during dialysis treatment. In CBT, ACT, self-regulation, and Dohsa-hou, it is necessary to focus on one's own experiences of direct control (Friedberg et al., 2012), development (Hayes et al., 2013), feedback control process (Carver and Sheier, 1998), and body movement processes (Naruse, 1988). In the setting of the present study, the participants had to engage in body awareness through Dohsa-hou techniques and define their self-awareness by means of the interview in terms of their physical conditions, particularly with regard to pain. In future research, it would be useful to investigate the interaction between chronic patients' self-awareness and various clinical effects of Dohsa-hou.

The present study used a collaborative management of chronic illness approach for low-functional HD patients with chronic pain that coordinated with other health care providers to enhance feasibility and adherence (Von Korff et al., 1997). Relaxation training is one of the most widely used and effective pain coping skills (Keefe et al., 2018). Dohsa-hou focuses on body movements that anyone can perform, with no particular emphasis on disorder, dysfunctions, or disabilities. It is a simple and versatile psychological treatment technique for health providers and could usefully be integrated through future research into other settings such as nursing care plans that incorporate range of motion exercises for treatment toolkits (Tseng et al., 2007; Gordon and Bloxham, 2016). The target of the body, relative ease of use, and straightforward psychoeducation associated with Dohsa-hou may serve as distinct advantages for holistically tackling cases of severe chronic pain, especially if it can be utilized as another relaxation-based skill available to buffer simultaneous treatment modalities (Barlow et al., 2002). Toward this end, and as a direct result of the current study, the health care staff under supervision successfully integrated and longitudinally implemented the Dohsa tasks throughout a 4 months period, completing and providing data for almost all of the 48 sessions. Along with the indicators of effectiveness from the pilot implementation, this record of completion with low attrition indicates strengths for the program in terms of procedural fidelity, applicability, and feasible transfer to more rigorous designs for future research.

## Limitations

It is important to note that the strength of these findings is tempered by the fact that it was a pilot implementation in a small number of cases with key representativeness and design limitations. Only 9 (13%) patients reported chronic pain when we interviewed possible patients for recruitment to this study. This differs from Brkovic et al. (2016) who reported that the general prevalence of chronic pain of HD patients ranged from 33 to 92%. As noted as a point of consideration in systematic guidelines such as the SCRIBE Checklist (Tate et al., 2016) and the Risk of Bis in *N*-of-1 Trials (RoBiNT) Scale (Tate et al., 2014), it should be emphasized that this study is a preliminary observation from one hospital and this discrepancy might limit the generalizability of these findings.

In addition, we cannot ignore the fact that positive outcomes could be explained by non-specific factors, such as the treatment expectancy effects of being in a clinical study and receiving extra attention from treatment staff. An especially notable limitation in our study is that our data cannot tease apart the effects of positive feedback itself. Improving communication skills (Ricka et al., 2002; Redondo et al., 2004), minimizing the burden for patients (Griffith and Gaby, 2005; Peterson, 2010), and engaging in voluntary activities by moderate requests could have played a role (Friedberg et al., 2012). These strategies have often been used to elicit effective cooperation from patients toward better pain relief outcomes and self-management. In addition, this study depended on the patients' self-evaluation of their own pain level and there was a lack of medical diagnostic evaluation explicitly incorporated into the dataset to account for confounders, a control group, or measurement of pain-related mediators (e.g., pain inventory, pain impairment belief, and psychological flexibility). In future research, the use of more detailed semi-structured interviews incorporating questionnaires such as the Brief Pain Inventory or Health-Related Quality of Life Measures is suggested to clarify or monitor the roles of pain intensity and or quality of life with greater specificity.

As this was a chiefly idiographic assessment of single-case series with a simple phase design using participants as their own control, the RoBiNT Scale as a resource indicates a number of other possible risks of bias that will need to be carefully addressed in future studies (Tate et al., 2014). While the length of the period of study provided a sufficient number of data points for inter-subject replication, it was still difficult to secure baseline data that could statistically verify stable pain conditions, as seen in the emergence of higher VAS pain scores for two of the cases. However, consistency of data patterns across the similar phases for the other three cases appears to be supported by visual inspection (Figure 4 and Table 3) and from the interview reports of continued use of the tasks at home (Table 2). The data collection was scheduled and did not commence at randomly assigned start points under blind conditions. While coping skill acquisition and psychoeducation are cumulative in ways that can make it challenging to organize certain manipulations, future studies with randomized single-participant designs perhaps utilizing alternating treatments or changing criterions could be used to build on the proof-of-concept and offer more to the credible evidence base for interventions for chronic pain in hemodialysis patients using Dohsa-hou (Tate et al., 2014; Morley, 2018).

In sum, although limited, in light of the feasibility indicators of procedural fidelity and proof-of-concept in a cohort of a longitudinally applied clinical context for five participants of a known group, some degrees of effectiveness of the intervention for the clinical end points were observed to allow for the stratification of future patient groups (Lillie et al., 2011). Future studies should build on this proof-of-concept trial by monitoring other factors that affect the condition of patients in terms of physical health status as well as individual differences in pain-related constructs. While meta-analyses have indicated that psychological interventions demonstrate efficacy for chronic pain, randomized controlled trials remain the level of rigor necessary to establish their efficacy (Veehof et al., 2011). Therefore, randomized controlled trials and studies with larger samples and a comparison with other psychotherapies are needed to confirm the validity of the present findings.

## Conclusion

The findings indicated areas of support in the domain of the promotion of self-regulation through Dohsa-hou as an adaptive pain coping skill. Specifically, this study suggested that applying body movement relaxation tasks shows promise as a psychotherapeutic approach for managing severe chronic pain, especially in a sample of individuals affected by renal disease. Dohsa-hou provided indicators of effectiveness by reducing pain during dialysis as well as improving quality of life through reports of recouped control over patient-driven desired commitments to personal care and domestic activities. Overall, this study suggested that hemodialysis patients are capable of learning to manage their chronic pain on their own through the deliberate practice of movement tasks aimed at changing feeling states and that a collaborative program to facilitate the care of chronic pain can be provided by clinical staff using Dohsa-hou. Although more research is needed, this study indicated that one way to facilitate quality of life change in low-functioning patients undergoing dialysis is to relieve the burden of chronic bodily pain by promoting self-management with body movement-based relaxation.

## Ethics Statement

This study was carried out in accordance with the recommendations of the Ethical Committee of the Graduate School of Education, Hiroshima University. The protocol was approved by the Ethical Committee of the Graduate School of Education, Hiroshima University. All subjects gave written informed consent in accordance with the Declaration of Helsinki.

## Author Contributions

YH conducted the research, supervised the progress of the patients, and served as the primary author of the manuscript. RK provided manuscript preparation, analysis, revisions, summary, and literature review. KA provided ratings for the self-regulation categories, analytical insights, and revisions. TY contributed to confirmation of the research protocol, analysis, and manuscript preparation.

### Conflict of Interest Statement

The authors declare that the research was conducted in the absence of any commercial or financial relationships that could be construed as a potential conflict of interest.
